# Molecular diagnostics in the management of chronic hepatitis C: key considerations in the era of new antiviral therapies

**DOI:** 10.1186/1471-2334-14-S5-S8

**Published:** 2014-09-05

**Authors:** Bryan Cobb, Gabrielle Heilek, Regis A Vilchez

**Affiliations:** 1 Roche Molecular Systems Inc., Pleasanton, California, USA; 2 Abbvie, Chicago, Illinois, USA

## Abstract

Molecular tests that detect and/or quantify HCV RNA are important in the diagnosis and management of patients with chronic hepatitis C (CHC) undergoing anti-viral therapy. The primary goal of anti-HCV therapy is to achieve a sustained virologic response (SVR) defined as "undetectable" Hepatitis C Virus (HCV) RNA in the serum or plasma at 12 to 24 weeks following the end of treatment.

HCV RNA viral load (VL) monitoring is used to guide treatment duration where decisions can be made on-therapy and to determine whether or not to stop therapy. In addition, clinicians determine treatment regimen and duration based on the HCV genotype (1-6) as well as the kinetics of HCV RNA levels.

As direct acting antivirals (DAA) have revolutionized hepatitis C treatment, they have also lead to new HCV RNA VL result interpretations. Further, the clinical decisions were different for pegylated-interferon/ribavirin (PEGα/RBV)+ boceprevir or telaprevir-containing regimens approved in 2011 (*e.g*. one requiring an additional 4 week "lead-in" with PEGα/RBV), each having different HCV RNA values for futility rules, created complexity in clinical decisions.

The future pegylated-interferon free DAA- regimens promise significantly improved cure rates along with fixed durations and simpler treatment rules. The intent of this article is to discuss the role of HCV RNA real-time PCR tests used in the management of CHC patients in the past and how this is likely to change in the era of interferon free DAA regimens.

## Background

Chronic HCV (CHC) infection is a global public-health problem, with approximately 170 million persons chronically infected [1] who are at an increased risk of morbidity and mortality [2] due to liver cirrhosis, hepatocellular carcinoma (HCC), and extra-hepatic complications that develop. The incidence of cirrhosis and HCC is projected to dramatically increase over the next decade in certain populations such as the U.S. "baby boomer" birth cohort.

With the development of interferon free, all oral, potent antiviral agents with less adverse effects, the need for screening individuals and successfully treating at-risk CHC patients becomes increasingly more important and possible.

## New screening recommendations

The CDC has previously recommended routine HCV screening for persons most likely infected with HCV based on the known epidemiologic risk factors [2] and has published guidelines for laboratory testing using HCV antibody and HCV RNA assays [3]. In 2012, the CDC amended testing recommendations to include one-time HCV testing for all persons born between 1945 and 1965 ("baby boomers") in the U.S. [3].

## The new role for HCV RNA tests in the aid in diagnosis of CHC patients

Screening for hepatitis C starts with anti-HCV antibody. The OraQuick HCV Rapid Antibody Test (OraSure Technologies) is a rapid assay for the presumptive detection of HCV antibody in finger stick capillary blood and venipuncture whole blood [4]. In the U.S., this test is approved for use in doctor's offices or clinics that able to use laboratory-based IVD tests [3]. Rapid tests are also available in Europe as well as other parts of the world.

The Recombinant Immunoblot Assay (RIBA) HCV 3.0 Strip Immunoblot Assay (Novartis Vaccines and Diagnostics) that was previously recommended for supplemental testing of blood samples after initial HCV antibody testing is no longer available or recommended.

**Figure 1:**
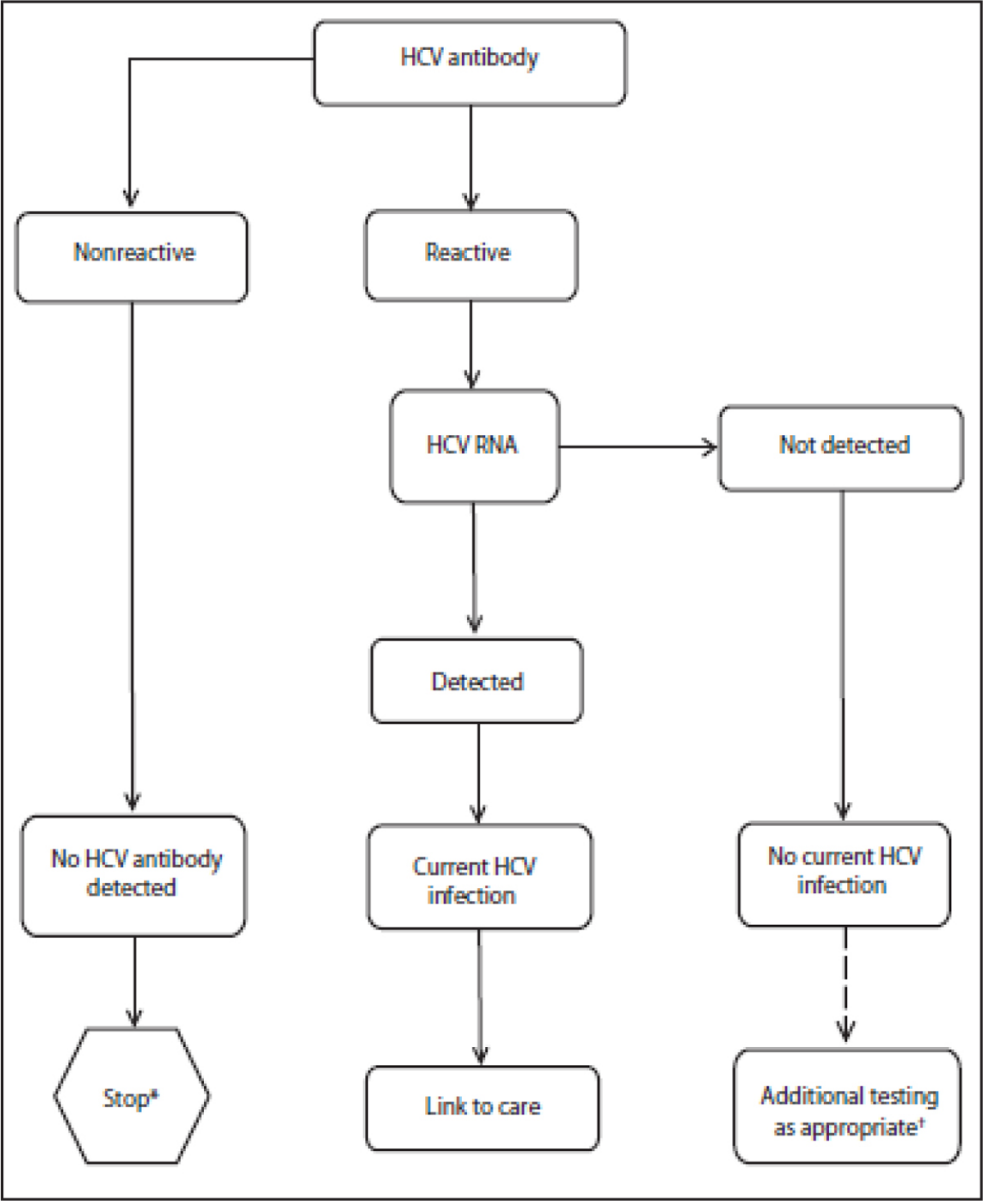
**Centers for Disease Control and Prevention recommendation: testing sequence for identifying hepatitis C virus infection**.

In 2013, the recommendations were updated for supplementary testing whereby the diagnosis of a current HCV infection (a positive antibody test) should be confirmed by using a NAT test (Figure 1). This is because an anti-HCV antibody test result can be positive in patients who were previously infected with HCV but have spontaneously cleared infection and are no longer viremic. HCV RNA tests can detect the presence of an active HCV infection. In clinical practice guidelines, using a sensitive molecular method (LLOD <15 IU/mL) is recommended for the diagnosis of acute hepatitis and CHC [4]. However, it's important to note that currently there are no real-time PCR HCV RNA viral load monitoring tests that have been reviewed or approved by any regulatory agencies, including the FDA that have a diagnostic intended use claim supporting these recommendations.

## Significance of VL monitoring

Measurement of HCV RNA is essential for measuring an active infection at baseline, during treatment, at the end of treatment, and for detecting relapse after stopping antiviral therapy (*e.g*. 12 or 24 weeks later). Absence of viral replication as measured in the bloodstream 3 or 6 months after an antiviral treatment regimen indicates the patient is cured.

### Current molecular methods

A variety of molecular methods have been used to manage CHC patients. The majority of tests that are used by routine clinical laboratories are based on real-time PCR technologies which quantify HCV RNA during the exponential phase of amplification, with greater sensitivity and a broader linear dynamic range (~10 to 10^8 ^IU/mL). There are several HCV RNA commercial, real-time PCR tests that are available (Table 1).

**Table 1:** Commercially available quantitative real-time PCR-based hepatitis C virus RNA assays

Assay	Vendor	Technology (target region)	IVD Approval Status	Dynamic Range (IU/mL)	LLOQ (IU/mL)	LLOD* (IU/mL)
COBAS^®^ Ampliprep/ COBAS^®^ TaqMan ^®^ v2.0 Test	Roche Molecular Systems	Real-time PCR, (5'UTR)	FDA, CE	15 to 1.00 × 10^8^	15	15
COBAS^®^ TaqMan ^®^ for use with the High Pure System Test, v2.0	Roche Molecular Systems	Real-time PCR, (5'UTR)	FDA, CE	25 to 3.91 × 10^8^	25	20
Abbott RealTi*m*e HCV Test	Abbott Diagnostics	Real-time PCR, (5'UTR)	FDA, CE	12 to 1.00 × 10^8 ^[27]	12	12
Versant HCV RNA Test, 1.0 (kPCR)	Siemens	Real-time PCR, (*pol *gene)	CE	15 to 1.00 × 10^8^	15	15
Artus Hepatitis C Test (QS-RGQ)	Qiagen	Real-time PCR (target proprietary)	CE	65 to 1 × 10^6 ^[9]	35	21

IVD: In vitro diagnostic. LLOQ: Lower limit of quantification. LLOD: Lower limit of detection (also referred to as analytical sensitivity). PCR: polymerase chain reaction. FDA: Food and Drug Administration. CE: European Community. HCV: Hepatitis C virus.

* LLOD shown is the overall analytical sensitivity, the LLOD by genotype and by matrix (plasma vs. serum) may be lower than the number shown.

The COBAS^® ^AmpliPrep / COBAS^® ^TaqMan^® ^HCV Quantitative and Qualitative Test, version 2.0 (TaqMan^® ^HCV Test, v2.0) (Roche Molecular Systems) uses a magnetic silica bead-based automated RNA extraction on the COBAS AmpliPrep platform followed by HCV target specific (5' UTR) amplification and detection performed on the COBAS TaqMan thermal cycler. The assay is approved as a FDA-IVD and CE-IVD quantitative test and as a CE-IVD qualitative test. Both the quantitative and qualitative tests use a dual probe approach where two fluorescently labeled hydrolysis probes simultaneously detect amplicon, providing broader detection and quantification of rare genotype 4 sequences [5,6]. HCV RNA titer is calculated using a competitive quantitative standard, obviating the need for the laboratory to perform calibrations. Reagents are stored at 2-8^o^C. A manual version, the COBAS^® ^TaqMan^® ^Test v2.0 for use with the High Pure System (the HP-TaqMan HCV, v2.0) (Roche Molecular Systems), which instead uses a column-based manual extraction is also available. This test has been predominantly used in the clinical trials for currently approved DAA-interferon containing regimens.

The Abbott RealTi*me *HCV assay (Abbott Molecular) uses an automated, magnetic particle-based nucleic acid extraction on the m2000sp platform followed by a manual sealing of the reaction plate to prepare it for HCV target specific amplification and detection on the m2000rt platform. To detect the HCV RNA target, a DNA probe with a covalently linked fluorescent moiety and a covalently linked quenching moiety is used. Since a noncompetitive internal control (derived from a pumpkin gene) is used, the laboratory is required to perform lot-specific calibrations. Reagents must be shipped and stored frozen [7].

The Versant HCV RNA 1.0 test (Siemens Healthcare) is a real-time PCR assay that uses a magnetic silica bead-based automated RNA extraction followed by automated amplification of the HCV genome and detection on the Versant Kinetic PCR (kPCR) Molecular System platform. This test replaces the quantitative, branched DNA (bDNA)-based, signal amplification test as well as the qualitative TMA-based test [8].

The Artus Hepatitis C QS-RGQ assay is a real-time PCR assay that uses a magnetic particle-based automated RNA extraction on the QIAsymphony SP platform (Qiagen) followed by amplification of the HCV genome and detection on the Rotor-Gene Q platform [9].

Other molecular methods that are used in management of CHC patients include genotyping tests (for HCV genotypes 1-6), which help determine the type and duration of treatment as well as to predict treatment outcomes. Currently, HCV genotyping tests use direct DNA sequencing (*e.g*. THE TRUGENE^® ^HCV Genotyping Assay, Siemens Erlangen, Germany) and bi-directional sequences where genotype and subtype characterization is determined by two fluorescently labeled DNA primers or a line probe assay (INNO-LiPA HCV II Genotype Test, Innogenetics, Ghent, Belgium), that simultaneously detects of 5′UTR and Core regions to improve genotype 1 characterization using a linear probe array [10].

Several real-time PCR-based non-IVD Tests (*e.g*. GenMark) are used and more recently the Abbott HCV Genotype Test is currently available as the only FDA-approved test. In a recent report, the Abbott HCV Genotype Test (Abbott Molecular) has been found to be useful for characterizing genotype 2-6 but may require a confirmatory method for correct genotype 1 characterization [11,12]

#### Non-molecular methods

HCV core antigen serology tests have been proposed for the use in either on-treatment monitoring or for assessing SVR, but this application may miss approximately half of the samples <2,000 IU/mL by PCR and may only be reliable in results >6,000 IU/mL [13]. Therefore, HCV core antigen may not be suitable for detecting an active HCV infection. Unlike HCV core antigen tests, the clinical utility of using HCV RNA PCR-based tests in managing CHC patients is well established [14].

## Treatment landscape; past, present, and future

After a decade of using PEG2α/RBV to treat CHC patients, boceprevir (VICTRELIS^®^, Merck & Co., Inc., Whitehouse Station, NJ) and telaprevir (INCIVEK^®^, Vertex Pharmaceuticals Incorporated Cambridge, MA), NS3/4A protease inhibitors, co-administered with PEG2α/RBV were approved for HCV genotype 1 infected patients in 2011 after demonstrating significant improvements in SVR rates.

Recently, at the end of 2013, two more drugs were approved demonstrating even greater improvements in SVR rates. Simeprevir (OLYSIO™, Janssen Therapeutics, Titusville, NJ) a NS3/4A protease inhibitor [15,16] and Sofosbuvir (SOVALDI™, Gilead Sciences, Inc., Foster City CA) a potent HCV nucleotide analog NS5B polymerase inhibitor are now available [15].

Simeprevir (plus PEG2α/RBV) was approved for HCV genotype 1 infected subjects with compensated liver disease (including cirrhosis) along with a screening requirement for patients with HCV genotype 1a infections for the presence of the NS3 Q80K polymorphism (in which case this therapy is not recommended).

Sofosbuvir represents the first all oral, interferon free DAA-containing regimen (combined with RBV) and the first DAA-interferon-free regimen approved for treating patients with HCV genotype 2 or 3 infections [17,18]. Sofosbuvir (plus RBV) has a shorter treatment duration for genotype 2 (12 weeks) than genotype 3 (24 weeks). HCV genotypes 1 or 4 infections can also be treated with sofosbuvir but require coadministration of PEGα/RBV for 12 weeks.

The AASLD/IDSA recommendations for testing, managing, and treating HCV were updated in 2014 in response to the changing landscape of HCV treatment options [19].

### HCV RNA Test results & interpretations

A definition and description of terms used to describe HCV RNA levels is provided (Table 2) and HCV RNA VL results and interpretations are described (Table 3).

**Table 2:** Definitions of key analytical performance terms used in defining hepatitis C virus RNA VL titer measurements based on guidelines [28]

Result	Definition
Target not detect	HCV RNA is not detected, no observable PCR amplification or detection
LLOQ	Lowest HCV RNA titer within the test's dynamic range that is quantifiable and accurate
LOD	Lowest amount of analyte in a sample that can be detected (*e.g*. Detection of HCV RNA ≥95% by Hit Rate or PROBIT analysis)
ULOQ	The highest HCV RNA titer result within the test's dynamic range that is quantifiable and accurate

HCV: Hepatitis C virus. LLOQ: Lower limit of quantification, LOD: Limit of detection (also referred to as "analytical sensitivity"). ULOQ: Upper limit of quantification

**Table 3:** The results and interpretations that are reported by manufacturers of commercial hepatitis C virus RNA VL tests

Titer result (IU/mL)	Reported results	Results interpretation*
"Target not detected" or "Not detected"	Results are reported as **"HCV RNA not detected"**.	Ct value for HCV is above the limit for the assay or no Ct value for HCV is obtained.
Less than the Lower Limit of Quantification (LLOQ)	Results are reported as **"HCV RNA detected, less than [*LLOQ*] IU/mL HCV RNA"**.	Calculated IU/mL is below the dynamic range of the assay.
Titer is within the linear range of the test	Results are reported as **"[*number*]** **IU/mL, HCV RNA detected"**.	Calculated results are quantifiable within the dynamic range of the test (*e.g*.greater than or equal to the LLOQ and less than or equal to ULOQ, results)
Greater than the upper limit of quantification (ULOQ)	Results are reported as **"greater than [*ULOQ*] IU/mL HCV RNA"**.	Calculated results are above the dynamic range of the assay.

* Ct = crossing point or crossing threshold, the value in which the PCR amplification is detected (sigmoidal curve)

HCV: Hepatitis C virus. LLOQ: Lower limit of quantification. ULOQ: Upper limit of quantification.

To note, if HCV RNA is detected by PCR (and lower than the linear range of the test), the result is reported by the software as "HCV RNA, detected less than the Lower Limit of Quantitation (LLOQ)", even if the actual VL titer is below the sensitivity or Limit Of Detection (LOD) of the test. Being able to "*detect*" HCV RNA that is below the LOD of the test may seem counterintuitive since it is typically presumed that if the actual HCV RNA titer is below the LOD then there is nothing there to "*detect*".

However, the LOD is defined and calculated by the tests ability to detect HCV RNA ≥95% of the time. This means that even at HCV RNA titers that are half the LOD, the PCR amplification may still detect HCV RNA ~50% of the time, in which case, the result will be reported as "HCV RNA detected, < LLOQ" if the HCV RNA is "detected".

### Viral kinetics and RGT

In patients treated with PEGα/RBV, the best predictor of an SVR was shown to be a rapid on-treatment HCV RNA decline to "undetectable" early in therapy [20]. To this end, a rapid virological response (RVR), or "undetectable" (*e.g*. <50 IU/mL) by 4 weeks of PEGα/RBV, has been used to determine eligibility for shortening therapy (*e.g*. 24 weeks versus 48 weeks, genotype 1).

#### New definitions for an "undetectable" HCV RNA VL

While the goal of treating CHC patients is to eradicate the infection as measured by an "undetectable" HCV RNA result, "undetectable" has evolved alongside the treatment algorithm. For PEGα/RBV therapy, an "undetectable" result was any result that is <50 IU/mL (Table 4).

**Table 4:** Comparison of therapies and key clinical decisions using hepatitis C virus RNA VL

Antiviral therapy	Geno-type	Response guided therapy				Definition of "undetectable" HCV RNA result to assess SVR (IU/mL)
		**On-therapy decision?**	**Responder time point (weeks)**	**HCV RNA result considered "undetectable" †**	**Treatment duration (weeks)**	

**PEGα/RBV**	1-6	YES	4 or 12	week 4, (RVR) <50 IU/mL or week 12, (partial responder) 2 log_10 _drop	24, 48, or 72	< 50
**PEGα/RBV +TVR**	1	YES	4 and 12	TND, both RGT timepoints	24 or 48	< 25
**PEGα/RBV +BOC**	1	YES	8 and 16**	TND, both RGT timepoints	28, 36, or 48	< 25
**PEGα/RBV +SIM**	1	YES*	12	< 25 IU/mL	24 or 48	< 25
**PEGα/RBV +SOF**	1	N/A	N/A	N/A	12	< 25
**SOF/RBV **	2, 3	N/A	N/A	N/A	12 or 24	< 25

* Fixed duration regimen, HCV RNA VL ≥25 IU/mL is a stopping rule.

** with a 4 week lead-in (PEG**α**/RBV)

† The test used was the COBAS^® ^TaqMan^® ^for Use with the High Pure System (LLOQ = 25 IU/mL, overall LOD = 20 IU/mL)

HCV: Hepatitis C virus. SVR: Sustained virologic response. PEG**α**: Pegylated interferon alpha. RBV: ribavirin. RVR: Rapid virological response. TVR: Telaprevir. TND: Target not detected. RGT: Response guided therapy. BOC: boceprevir. SIM: Simeprevir. SOF: Sofosbuvir.

In contrast, for PEGα/RBV + boceprevir or telaprevir regimens, the term "undetectable" was defined as a "target not detected" result, which was required for patients to be eligible for shorten therapy; but for SVR assessments, a "<25 IU/mL, HCV RNA detected" was an acceptable endpoint.

For the recently approved regimen containing simeprevir, a stopping rule "cutoff" of 25 IU/mL is used at 4, 12, or 24 weeks in which all therapies are discontinued if HCV RNA results are above this cutoff (Table 4). For sofosbuvir, HCV RNA testing is only recommended after treatment after a fixed duration and to assess SVR. Both regimens use "<25 IU/mL, HCV RNA detected" for defining "undetectable".

In the "real world setting" it is likely that there will be less patient compliance than in the clinical trials. Therefore, it may be useful to investigate whether HCV RNA VL "adherence monitoring" on-therapy is worthwhile in patients suspected of noncompliance, especially when considering the high treatment costs.

Given that the trials used a test with a LLOQ of 25 IU/mL, differences in a tests LLoQ is important. How should clinicians handle a quantifiable result of 22 IU/mL derived from a different test than the one used in the clinical trials (*e.g*. one that has a lower LLOQ)? These are practical considerations that may cause uncertainty for clinicians.

#### Using "target not detected" for shortening therapy

With the introduction of boceprevir and telaprevir, new RGT rules were introduced which lead to considerable confusion in the terms used to define "undetectable" and when to apply this interpretation. These rules were based on a re-analysis of the boceprevir and telaprevir trials data that was published by the FDA where it was concluded that a "HCV RNA detectable, <LLOQ" result predicted a significantly lower cure rate compared with subjects with an "undetectable" ("Target Not Detected") result [21]. Based on this analysis, it was determined that a confirmed "detectable but below the LLOQ" HCV RNA result should not be considered equivalent to an "undetectable" HCV RNA ("Target Not Detected") result for the purposes of RGT. Therefore, a "Target Not Detected" result at both 4 and 12 weeks of PEGα/RBV + telaprevir therapy was required to shorten therapy (48 weeks to 24 or 36 weeks of PEGα/RBV). To further add complexity, stopping rules were also different for boceprevir and telaprevir regimens (100 and 1,000 IU/mL, respectively).

#### Differences between HCV RNA assays with DAA therapies

Although all commonly used HCV RNA assays report the results in the standardized IU/mL, not all tests perform similarly. Several reports have demonstrated differences between how assays report results, particularly in detecting low amounts of HCV RNA [22,23].

In these studies, concordance analyses have determined that HCV RNA differences in reporting results that are "Target Not Detected" versus "HCV RNA detected, < LLOQ" have become apparent.

This is particularly true in one study that investigated results generated from the TaqMan ^® ^HCV Test, v2.0 used as part of a phase III clinical trial with simeprevir plus PEGα/RBV and compared it to Abbott RealTi*m*e HCV Test [24]. Overall, there was good agreement between the 2 assays; however, a large number of samples (26%-35%) at week 4 of treatment had detectable HCV RNA levels (<LLOQ) with the Abbott RealTi*m*e assay that were "Target Not Detected" by the HPS-TaqMan ^® ^HCV Test, v2.0. These patients received shortened therapy based on the HPS-TaqMan ^® ^HCV Test, v2.0 TND result and high SVR rates were achieved. Thus, if the Abbott RealTi*me *assay results at week 4 of therapy had been used to determine treatment duration, these patients may have been over-treated by an additional 6 months.

Since these DAA-containing triple therapy requires HCV RNA to be TND at both weeks 4 and 12 in order to shorten therapy, differences between HCV RNA assays can affect key medical decisions, in this case resulting in a larger portion of patients treated for longer durations (if the same cutoffs are used). It was therefore suggested that a cutoff of <12 IU/mL (detected) may be appropriate for the Abbott RealTi*m*e HCV Test. However, since this cutoff has not yet been clinically validated and further studies are needed.

While boceprevir and telaprevir containing regimens have been replaced by more potent regimens, differences in the performance of HCV RNA tests might be of importance, particularly if they are not clinically validated.

#### Therapies expected in the near future

Faldaprevir is a HCV protease inhibitor in late stage phase 3 clinical trials and administered once daily is being tested in combination with PEGα/RBV, and in IFN-free regimens with other DAA agents.

Sofosbuvir is also being investigated in combination with antiviral agents that target different virus proteins such as daclatasvir and ledipasvir (nonstructural protein 5A [NS5A] inhibitors), with or without RBV [25]. Preliminary results of Phase 3 trials of the interferon-free sofosbuvir and ledipasvir combination regimen in patients with HCV genotype 1 infection have shown SVR12 rates of 93%-99% (Table 5).

**Table 5:** Summary of phase 3 trials of sofosbuvir and ledipasvir in hepatitis C virus genotype 1

Study	Population	Treatment/Duration	SVR12 rates
**ION-1 **	HCV GT1 treatment-naïve including 15.7% (136/865) with cirrhosis	SOF/LDV, 12 weeks	97.7% (209/214)
		SOF/LDV + RBV, 12 weeks	97.2% (211/217)
		SOF/LDV, 24 weeks	NA (n = 217)
		SOF/LDV + RBV, 24 weeks	NA (n = 217)

**ION-2 **	HCV GT 1 treatment-experienced including 20.0% (88/440) with cirrhosis	SOF/LDV, 12 weeks	93.6% (102/109)
		SOF/LDV+RBV, 12 weeks	96.4% (107/111)
		SOF/LDV, 24 weeks	99.1% (108/109)
		SOF/LDV+RBV, 24 weeks	99.1% (110/111)

**ION-3 **	HCV GT1 treatment-naïve	SOF/LDV, 8 weeks	94.0% (202/215)
		SOF/LDV + RBV, 8 weeks	93.1% (201/216)
		SOF/LDV, 12 weeks	95.4% (206/216)

SVR: Sustained virologic response. HCV: Hepatitis C virus. GT: Genotype. SOF: Sofosbuvir. LDV: ledipasvir. RBV: ribavirin. NA: not available.

Abbvie is evaluating an interferon-free 3-DAA combination regimen containing the ABT-450, ritonavir, and ABT-267 co-formulated tablet (ABT-450/r/ABT-267) and ABT-333 tablet administered with or without RBV. ABT-450 is a NS3A protease inhibitor; ABT-267 is a NS5A inhibitor; and ABT-333 is a non-nucleoside inhibitor of the NS5B polymerase. This 3-DAAs regimen with and without RBV has reported SVR12 rates of 90%-100% in a phase 2 trial of patients infected with HCV genotype 1 [26]. Preliminary results of Phase 3 trials of this 3-DAA regimen have shown very high SVR12 across different HCV genotype 1 infected patient populations (Table 6).

**Table 6:** Summary of phase 3 trials of ABT-450/r/ABT-267, ABT-333 plus ribavirin in hepatitis C virus genotype 1

Study	Population	Treatment/Duration	SVR_12_
**PEARL-II**	GT1b treatment-experienced (N = 179)	3-DAA + RBV, 12 weeks (n = 88)	97%(85/88)
		3-DAA only, 12 weeks (n = 91)	100% (91/91)

**PEARL-III**	GT1b treatment-naive (N = 419)	3-DAA + RBV, 12 weeks (n = 210)	99% (209/210)
		3-DAA only, 12 weeks (n = 209)	99% (207/209)

**PEARL-IV**	GT1a treatment-naive (N = 305)	3-DAA + RBV, 12 weeks (n = 100)	97% (97/100)
		3-DAA only, 12 weeks (n = 205)	90% (185/205)

**TURQUOISE-II**	GT1 treatment-naive and treatment-experienced with compensated cirrhosis (N = 380)	3-DAA + RBV, 12 weeks (n = 208)	92% (191/208)
		3-DAA + RBV, 24 weeks (n = 172)	96% (165/172)

**SAPPHIRE-I**	GT1 treatment-naive (N = 631)	3-DAA + RBV, 12 weeks (n = 473)	96% (455/473)

**SAPPHIRE-II**	GT1 treatment-experienced (N = 394)	3-DAA + RBV, 12 weeks (n = 297)	96% (286/297)

SVR: Sustained virologic response. GT: Genotype. DAA: Direct acting antivirals. RBV: ribavirin.

## Future directions

Given the global burden of CHC and the advent of newer, more potent regimens with higher cure rates, increasing screening to identify at-risk CHC patients and linking them to care is even more important. New guidelines that support screening are important but linkage to care is an ongoing global challenge.

With the first DAA-containing regimens, clinical decisions based on HCV RNA VL results (and new interpretations) created complexity for the laboratory and clinician. Further, tests were shown to perform differently in some of DAA-containing regimens. Therefore, additional testing with each new DAA containing regimen across various commercially available HCV RNA tests is important.

While the new interferon-free therapies have demonstrated greater efficacy, accurate HCV RNA quantification remains important. In addition, interferon-free regimens may have fixed durations, but on-therapy "adherence monitoring" may be helpful, particularly given the high cost of the new regimens. Therefore, for these and other reasons discussed here, measuring HCV RNA will likely continue to be important.

## List of abbreviations

CHC: chronic hepatitis C; CLIA: Clinical Laboratory Improvements Amendment; DAA: direct acting antivirals; HCC: hepatocellular carcinoma; HCV: hepatitis C virus; IU: international units; LLOD: lower limit of detection; LLOQ: lower limit of quantitation; LOD: limit of detection; NAT: nucleic acid amplification; NS5A: nonstructural protein 5A; PCR: polymerase chain reaction; PEGα/RBV: pegylated-interferon/ribavirin; RGT: response-guided therapy; RVR: rapid virological response; SVR: sustained virologic response; ULOQ: upper limit of quantitation; TMA: transcription mediated amplification.

## Competing interests

Bryan Cobb is an employee of Roche Molecular Systems Inc.

Regis A. Vilchez is an employee of Abbvie

## Authors' contributions

BC, GH and RV contributed to the data analysis and manuscript writing.

## Declarations

B. Cobb and G. Heilek are employees of Roche and provided a sponsorship for this supplement. COBAS TaqMan tests referred here were developed by Roche. Vilchez is an employee of Abbvie and has not provided a sponsorship for this supplement.

This article has been published as part of *BMC Infectious Diseases *Volume 14 Supplement 5, 2014: Proceedings of the Third Workshop of the HCV Study Group in the Calabria Region: Results from the South Italian Network for Rational Guidelines and International Epidemiology (SINERGIE) Project. The full contents of the supplement are available online at http://www.biomedcentral.com/bmcinfectdis/supplements/14/S5.
